# Macrophage IL-1β turns meningeal fibroblasts into inflammatory amplifiers in pneumococcal infection

**DOI:** 10.3389/fimmu.2026.1808185

**Published:** 2026-05-14

**Authors:** Paul Beckenbauer, Linda Ercegovac, Greta Christensen, Pia Lagler, Sven Hammerschmidt, Stefanie Völk, Hans-Walter Pfister, Matthias Klein, Uwe Koedel, Susanne Dyckhoff-Shen

**Affiliations:** 1 Department of Neurology, Ludwig-Maximilians-University (LMU) University Hospital, Ludwig-Maximilians-University (LMU) Munich, Munich, Germany; 2 Department of Molecular Genetics and Infection Biology, Interfaculty Institute for Genetics and Functional Genomics, Center for Functional Genomics of Microbes, University of Greifswald, Greifswald, Germany

**Keywords:** cytokines, IL-1β, IL-6, macrophages, meningeal fibroblasts, neuroinflammation, pneumococcal meningitis, *Streptococcus pneumoniae*

## Abstract

**Introduction:**

In pneumococcal meningitis, a massive inflammatory reaction is triggered by the host immune system, leading to neurological damage. However, the mechanisms underlying the initiation and regulation of this response, particularly by resident cells, remain incompletely understood. Despite their strategic localization at the host-pathogen interface, the role of meningeal fibroblasts in pneumococcal meningitis remains poorly defined. This study therefore aimed to investigate their contribution to the immune response against *Streptococcus pneumoniae*.

**Methods:**

Primary meningeal fibroblasts were exposed to *Streptococcus pneumoniae*, and their cytokine responses were quantified in monoculture and in co-culture with macrophages using both direct contact and indirect systems.

**Results:**

Meningeal fibroblasts responded to pneumococcal challenge by producing a selective set of cytokines. This activation occurred independently of Toll-like receptor signaling. In co-culture, macrophages markedly enhanced fibroblast-derived cytokine production (including IL-6, IL-8, and CCL2) in both direct and indirect systems, indicating a robust amplification of the immune response. Mechanically, this effect was driven by macrophage-derived IL-1β, which we identified as the key factor of meningeal fibroblast activation.

**Discussion:**

These findings establish an IL-1β-driven macrophage-fibroblast axis as a key driver of inflammatory amplification in pneumococcal central nervous system infection and suggest a tractable target for therapeutic intervention.

## Graphical Abstract

**Figure:**
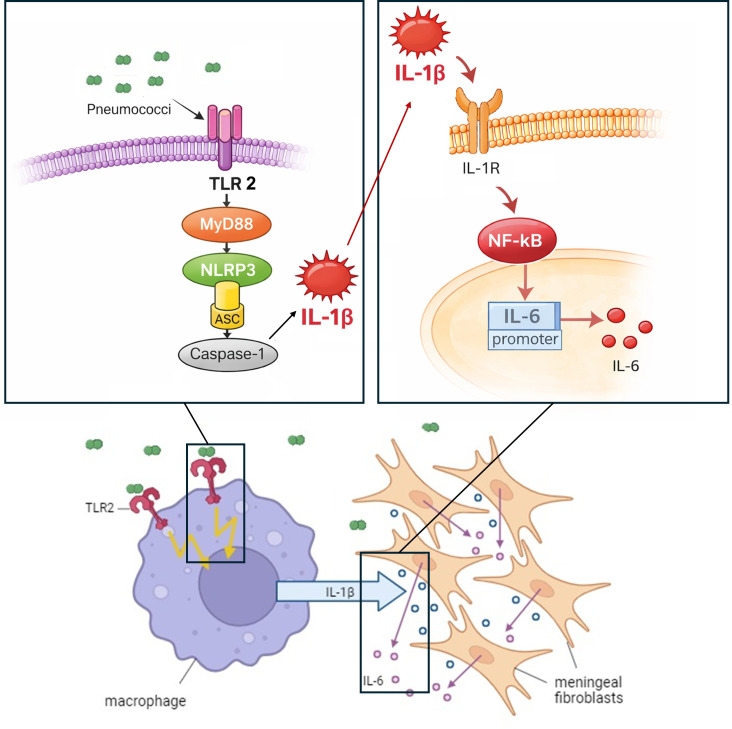

## 1 Introduction

Bacterial meningitis remains a major global health threat, associated with high mortality and morbidity (1). *S. pneumoniae* (SP) is the leading cause of bacterial meningitis in Europe, with a case fatality rate of 10-20% (2). Pneumococcal infection of the leptomeningeal space triggers a powerful inflammatory response that substantially contributes to brain damage and unfavorable outcomes (2). While the molecular mechanisms involved in recognizing SP have been extensively studied, the specific cell populations within the leptomeningeal space responsible for its detection and the initiation of the immune response remain elusive.

Macrophages, as resident immune cells, have been proposed as central players and have been extensively studied. However, the depletion of meningeal macrophages using CI2MDP liposomes resulted in surprisingly mild and heterogeneous effects on the inflammatory response. In a rabbit model of pneumococcal meningitis (PM), macrophage depletion by CL2MDP liposomes did not significantly attenuate inflammation in the subarachnoid space (3). In a rat model, depletion of macrophages using the same approach led to increased bacterial counts in the cerebrospinal fluid (CSF) and blood, decreased leukocyte influx into the CSF, and enhanced MIP-2/CXCL2 levels in the CSF (4). Similarly, in mice treated with CL2MDP liposomes and infected with SP, bacterial loads in the meninges and brain were elevated, whereas recruitment of immune cells - including neutrophils, monocytes, B cells, and T cells - as well as cytokine and chemokine expression were reduced in the meninges (5). In our murine model, depletion of meningeal macrophages using CL2MDP liposomes resulted in increased bacterial titers, accompanied by elevated levels of pro-inflammatory cytokines and chemokines in the brain (6). Together, these results suggest involvement of additional local cells in triggering the immune response to pneumococcal infection of the CSF.

Previously, we investigated the role of other cell types, such as mast cells and pericytes, in PM. Both cell types released selective cytokines upon pneumococcal exposure *in vitro*. While mast cell deficiency did not affect disease severity *in vivo* (7), pericyte depletion resulted in increased leukocyte infiltration and a worsened disease course, suggesting a protective role for pericytes in pneumococcal meningitis (8).

Meningeal fibroblasts represent the largest cell population within the leptomeningeal space, alongside resident immune cells and other stromal cells (9). Their role in health and disease remains incompletely characterized. They contribute to the formation of glia limitans and basement membrane and interact with dural and perivascular macrophages (10, 11). Previous studies suggest a role of CNS fibroblasts in stroke and spinal cord injuries (12). During neuroinflammation, fibroblasts participate in the formation of a fibroblastic reticular network (13) which appears beneficial in recovery from viral infection but detrimental in demyelination conditions; yet the underlying signaling mechanisms remain unclear (13). Moreover, CNS fibroblasts are involved in fibrotic scarring after injury or inflammation (13) and have been reported to participate in the clearance of bacteria, apoptotic cells, and neurotoxic peptides from the CSF (14, 15). Additionally, they can secrete cytokines in response to stimulation with phorbol 12-myristate 13-acetate (PMA) or *Escherichia coli* lipopolysaccharide (LPS) (16, 17).

Previous *in vitro* studies have explored the role of meningeal fibroblasts in bacterial CNS infections, primarily using human meningioma cells as a surrogate model. These cells exhibit robust cytokine and chemokine responses to pathogens such as *N. meningitidis* and *H. influenzae*. In contrast, SP has not been shown to induce cytokine secretion in this model, despite causing cell death at high bacterial concentrations (18–20). These limited and partly conflicting observations highlight a critical gap in our understanding of fibroblast responses to pneumococcal infection. To address this, we investigated the effects of pneumococcal challenge using both the established meningioma cell line Ben-Men-1 (16) and primary human meningeal fibroblasts (HMC) as a more physiologically relevant model. To our knowledge, this is the first study to systemically characterize the inflammatory responses of primary meningeal fibroblasts to SP.

## 2 Materials and methods

### 2.1 Cell culture experiments

Two cell models were used to study meningeal fibroblast responses: (i) the human meningioma cell line Ben-Men-1 was employed to enable comparison with previous studies using meningioma cell lines, and (ii) primary human meningeal fibroblasts (HMC) were used as a more physiologically relevant model.

Ben-Men-1 cells were cultured in T75 flasks in Dulbecco’s Modified Eagle’s Medium(DMEM) with 10% fetal bovine serum (FBS) and 10µg/ml penicillin/streptomycin (P/S) (Sigma-Aldrich) (21). Cells were sub-cultivated every 7 days by washing with PBS, harvesting with trypsinization, centrifuging and reseeding at 0.6 x 10^6^ cells in another T75 flask in 25ml medium. Stimulation experiments were conducted in DMEM with 10% FBS and P/S. In a subset of experiments, Ben-Men-1 cells were cultured for 7 days in meningeal cell medium (MCM, 2% FBS, 10µg/ml P/S, 1% meningeal cell growth supplement [MCGS, ScienCell]) in poly-l-lysine (PLL, ScienCell) coated flasks prior to stimulation in MCM with 1% FBS and P/S. Cells were then harvested and seeded into cell culture plates (direct co-culture: 3x10^5^ cells/well with 3x10^4^ THP-1/well in 24-well plates, indirect co-culture with conditioned supernatant: 5x10^4^ cells/well in 96-well plates), with cell numbers adjusted to the respective growth area. Additionally, selective stimulation experiments were conducted in human CSF collected from patients with idiopathic intracranial hypertension (IIH) or normal pressure hydrocephalus (NPH) undergoing CSF drainage (**Supplementary Table 1**).

HMC (#1400, ScienCell) were cultured in MCM with 2% FBS, 10µg/ml P/S and 1% MCGS (ScienCell) in PLL-coated T75 flasks. At 95% confluency, HMC were harvested by trypsinization and seeded in a microwell plate for pneumococcal stimulation experiments (25,000 or 50,000 per well). In direct co-culture experiments, macrophages were added after 48h, and the medium was replaced with stimulation medium (MCM, 1% FBS, 10µg/ml P/S, 1% MCGS).

The human monocyte cell line THP-1 was cultured in RPMI 1640 supplemented with10% FBS and 10µg/ml P/S (Sigma-Aldrich) in T25 flasks and passaged every 3–4 days. For differentiation into macrophages, THP-1 cells were treated with 100ng/ml PMA for 24h and left to rest without PMA for another 24h before being harvested with Accutase^®^ and seeded into microwell plates for subsequent experiments. For direct co-culture with Ben-Men-1 cells, 3x10^4^ THP-1 cells per well, suspended in MCM, were added to the 24-well plates. Wild-type as well as TLR2^-/-^, ASC^-/-^ and NLRP3^-/-^ THP-1 macrophages were purchased from Invivogen (thpd-kotlr2, thp-koascz and thp-konlrp3z).

Peripheral blood mononuclear cells (PBMC) were isolated from blood samples of healthy volunteers. Briefly, 20ml of whole blood was layered onto 20ml Histopaque-1077 (Sigma-Aldrich) and centrifuged at 400xg for 30min. The opaque fraction was collected, followed by lysis of contaminating erythrocytes using a hypotonic (0.2%) NaCl solution, and washing steps with PBS. The obtained cell pellet was resuspended in stimulation medium (MCM, 1%FBS, 10µg/ml P/S, 1% MCGS) and cells were identified as monocytes by their morphological characteristics using a phase contrast microscope. PBMC were subsequently used directly for pneumococcal stimulation experiments in analogy to THP-1-derived macrophages. All cells were cultured at 37 °C in 5% CO_2_.

### 2.2 Pneumococcal strains used in infection experiments

*S. pneumoniae* serotype 2 strain D39 (NCTC 7466; SP2), serotype 7F (SP7F; blood isolate, kindly provided by Gregor Zysk, Düsseldorf, Germany), serotype 6B (SP6B, kindly provided by Jeffrey Weiser, NYU, USA) and pneumolysin (PLY)-deficient mutant D39Δ*ply* (22) were grown on Columbia blood agar plates (Thermo Scientific, Germany) at 37 °C and 5% CO_2_ for 8–10 h. To prepare infection doses, pneumococci were cultured in Todd-Hewitt broth (THB; Roth, Germany) supplemented with 0.5% yeast extract (THY) and cultivation was performed at 37 °C in a water bath without agitation. After reaching an OD_600_ of 0.35 to 0.4, equivalent to the mid-log exponential growth phase, bacteria were harvested, resuspended in THY containing 20% glycerol and adjusted to 1x10^9^ bacteria per ml for cryo-conservation at -80 °C. Prior to infection experiments the colony forming units (CFU) were approved and host cells infected with the appropriate CFU mentioned in the experiments.

### 2.3 Co-cultures

Co-cultures of meningeal cells and macrophages were performed either as (i) direct co-culture in 96-well or 24-well plates allowing cell-cell-contact (macrophage-fibroblast ratio 1:10) or as indirect co-culture. Indirect co-culture was conducted either (ii) using HTS Transwell plates (Corning), in which cells were separated by a 3µm pore membrane, or (iii) by adding 10% conditioned supernatant from SP-stimulated macrophages (5x10^4^ or 1x10^5^ cells/well, 3x10^7^ CFU/ml (MOI: 60, 120), 24h) to meningeal cells (Ben-Men-1: 0.5x10^7^ CFU/ml, MOI: 20, 24h) during stimulation. Pure THY medium without bacteria served as a negative control.

In (co-)stimulation experiments, cells were then exposed to SP (serotype 2, D39, NTCT 7466) at concentrations of 0.25-8x10^7^ CFU/ml (MOI: 10-320) for 6h or 24h. In selected experiments, the PLY-deficient mutant D39Δ*ply* or additional pneumococcal serotypes (6B, 7F) were used. Prior to stimulation, pneumococci were lysed with 10% P/S to ensure consistent bacterial counts and release of pathogen-associated molecular patterns (PAMPs), thereby reflecting clinical conditions. THY medium without bacteria was used as a control.

### 2.4 Inhibitors

To investigate signaling pathways involved in SP-induced cytokine production, cells were pretreated with pharmacological inhibitors targeting transcriptional regulators implicated in IL-6 expression, based on current literature, including Nuclear factor-κB (NF-κB), cAMP-responsive element-binding protein (CREB), Activator protein 1 (AP-1) and bromodomain-containing protein 4 (BRD4) from the family of Bromodomain and extra-terminal domain (BET) proteins (**Supplementary Figure 1**) (23–26). Several inhibitors were used in stimulation experiments: NF-κB inhibitors BAY 11-7082 (0.1-10µM, Hycultec, Germany) and parthenolide (0.5µM Cayman Chemical, USA), BRD4 inhibitors JQ1 (0.01-1µM, Hycultec, Germany) and I-BET (10µM, Selleckchem, USA), CREB inhibitors KG-501 (10µM, Selleckchem, USA) and 666-15 (0.01-1µM, Tocris Bioscience, UK) and selective inhibitor of transcription factor AP-1 SR11302 (1µM, Tocris Bioscience, UK). In addition, TLR signaling was assessed using an anti-TLR2 mAB T2.5 (25µg/ml, Hycult), TLR4 inhibitor TAK-242 (5µM, Calbiochem, USA) and chloroquine (20µg/ml, Sigma-Aldrich, USA) to inhibit endosomal TLRs (**Supplementary Figure 1**). Controls were either PBS or DMSO in respective dilution.

**Figure 1:**
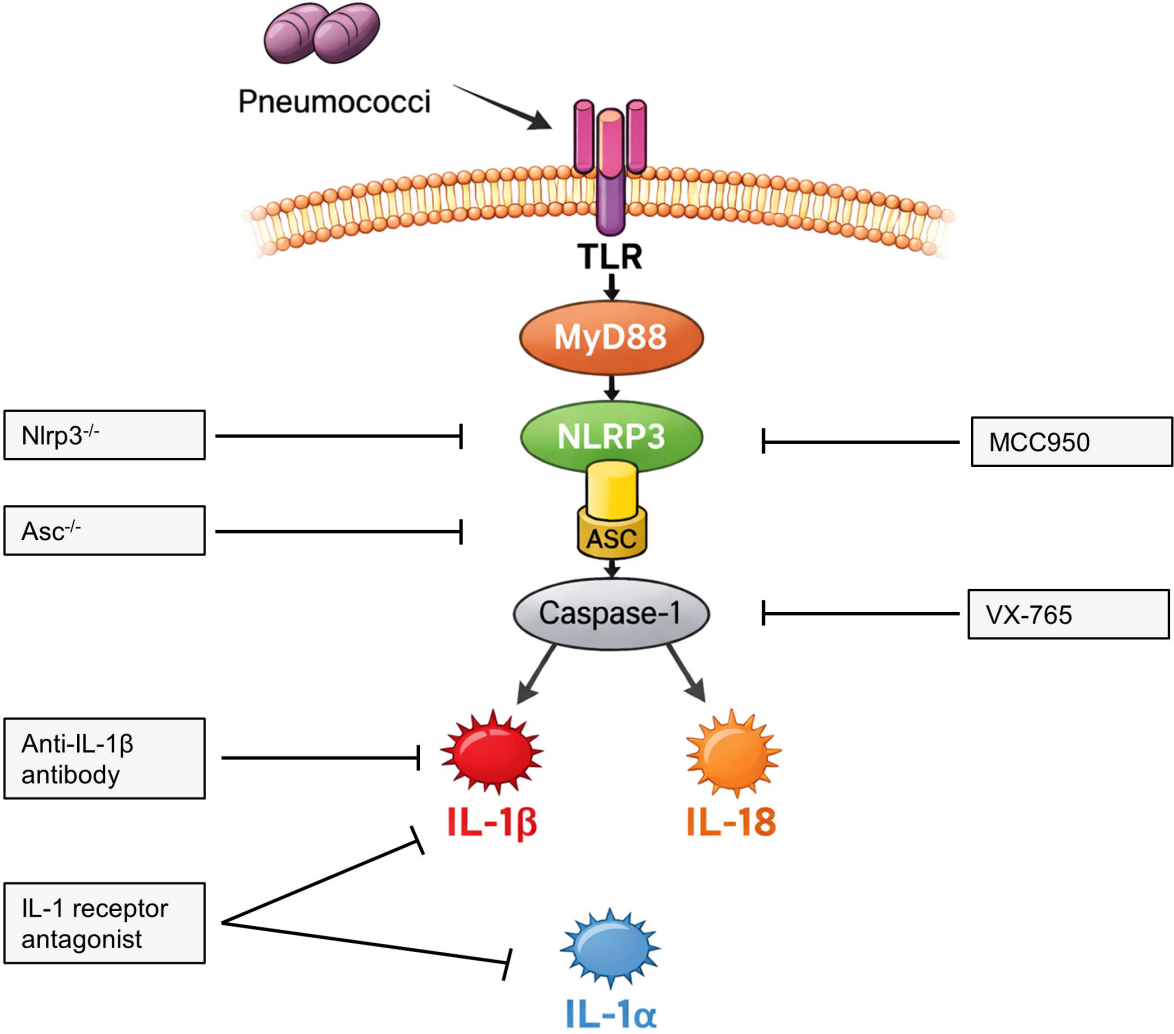
Experimental design to identify IL-1β as the key mediator of meningeal fibroblast activation during pneumococcal infection. Schematic overview of pneumococcal sensing and inflammasome activation in macrophages. TLR signaling via MyD88 leads to activation of the NLRP3 inflammasome and caspase-1–dependent processing of IL-1β and IL-18. Genetic and pharmacological approaches were used to inhibit inflammasome signaling, and IL-1 receptor blockade and IL-1β neutralization were applied to identify IL-1β as the key mediator.

To identify the mediator responsible for meningeal cell activation within pneumococci-stimulated THP-1 macrophage supernatant, supernatants were heat-inactivated (60 °C, 30min) or forced through 3kDa or 50kDa filters prior to application to meningeal fibroblasts. In additional experiments, THP-1 macrophages were treated with caspase-1 inhibitor VX-765 (100µM) or NLRP3 inhibitor MCC950 (10µM) (SelleckChem, Catalog No. S2228 and S7809, respectively) during pneumococcal challenge. To confirm the relevance of IL-1β, meningeal cells were treated with IL-1β antibody (25µg/ml, control: 25µg/ml mouse IgG1 control antibody; R&D Catalog No. MAB401 and MAB005, respectively) or IL-1 receptor antagonist anakinra (5µg/ml, MedChemExpress Catalog No. HY-P7029, control: endotoxin-free water + 0.1% FBS) additionally to conditioned supernatant from stimulated macrophages (**Figure 1**).

### 2.5 Determination of cell death

Lactate dehydrogenase (LDH) was determined as indicator of macrophage cell death in cell culture supernatants collected 6h or 24h after pneumococcal challenge (Biovision Inc., Roche).

### 2.6 Measurement of cytokines in cell culture supernatants

Concentrations of IL-1β, IL-6, IL-8 and CCL2 in human cell culture supernatants were determined by ELISA (R&D Systems) according to the manufacturer’s instructions. Additional cytokines, including CXCL1 or CCL2, were analyzed using a Proteome Profiler Array (R&D Systems).

### 2.7 PCR

After pneumococcal challenge and magnetic cell separation, cells were lysed and frozen at -80 °C. RNA was isolated using the Aurum^®^ Total RNA Mini Kit (#732-6820, Bio-Rad) and the amount of isolated RNA was measured with the Qubit^®^ RNA BR Assay Kit (#Q10211; Invitrogen). A defined amount of RNA (1µg) was converted into cDNA by iScript cDNA synthesis Kit (#1725034, Bio-Rad). PCR was performed with Real-Time rapid PCR qTower (Jena Analytics), PrimePCR™ primers assays for IL-1β, TLR2, fibronectin, CD45, IL-1 receptor (IL-1R), IL-6, PDGF-receptor α, PDGF receptor β and collagen1A1 (qHsaCID0022272, qHsaCED0036567, qHsaCED0043611, qHsaCED0038908, qHsaCIP0027424, qHsaCED0044677, qHsaCID0013272, qHsaCID0007202, qHsaCED0002181, Bio-Rad) and Sso Advanced Universal SYBR^®^ Green Supermix (#1725270, Bio-Rad) according to the manufacturer’s instructions. Gene expression levels were normalized to the house-keeping gene GAPDH (qHsaCED0038674, Bio-Rad), which showed stable expression across all experimental groups.

### 2.8 Statistical analysis

Experiments were generally performed with at least two independent biological replicates, each measured in technical triplicates, unless stated otherwise. Statistical analysis was performed using GraphPad Prism. The principal statistical test was one way analysis of variance and subsequent Tukey *post-hoc* tests. Differences were considered significant at *p* < 0.05. Data are displayed as means ± standard deviation (SD) as well as individually as circles/dots.

## 3 Results

### 3.1 Culture conditions modulate Ben-Men-1 cell responses to *S. pneumoniae*

**Figure 2:**
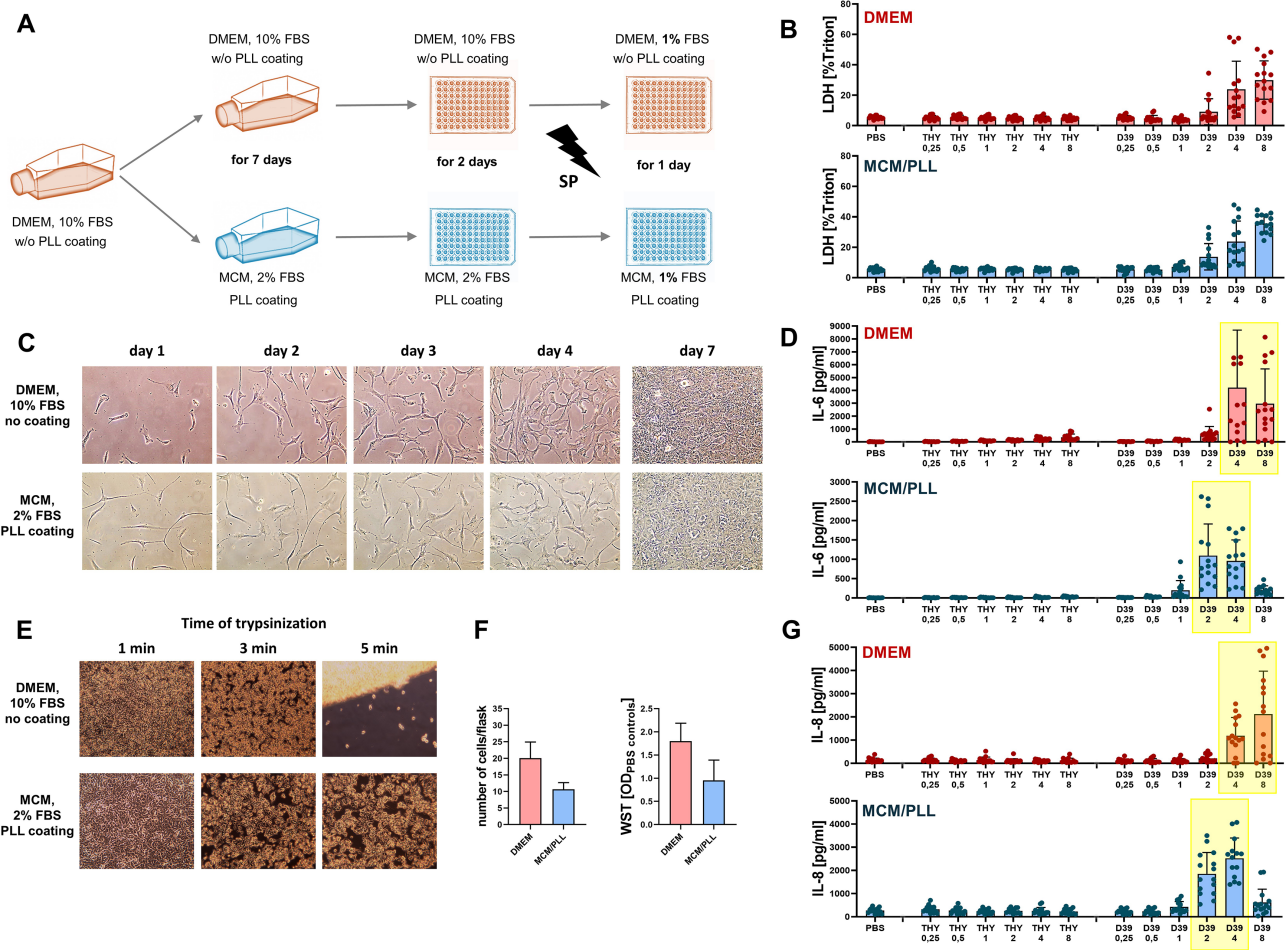
Phenotype, growth and cytokine release upon pneumococcal exposure of Ben-Men-1 cells depends on cell culture conditions. Ben-Men-1 cells were cultured for 7 days either in DMEM with 10% FBS w/o coating or in MCM with 2% FBS and PLL-coating, cultured in a microtiterplate for 2 days in respective medium and challenged with SP D39 for 24h in respective medium with 1% FBS **(A)**. During 7 day culturing in either DMEM or MCM, cells show different growth rates and phenotypes **(C)**. Trypsinization removed cells quicker after 5min when cultured in DMEM without coating than in MCM with PLL-coating **(E)**. After 7 days of culturing in DMEM vs. MCM, cell count and WST is higher in DMEM-cultured cells **(F)**. After challenge with SP in different concentrations (0.25x10^7^, 0.5x10^7^, 1x10^7^, 2x10^7^, 4x10^7^ or 8x10^7^ CFU/ml), cell culture supernatants were analyzed for LDH release **(B)**, IL-6 release **(D)** and IL-8 release **(G)**. Negative control was performed with pure bacterial THY growth medium without pneumococci in respective dilutions or PBS. Data are given as individual values as well as means ± SD.

**Figure 3:**
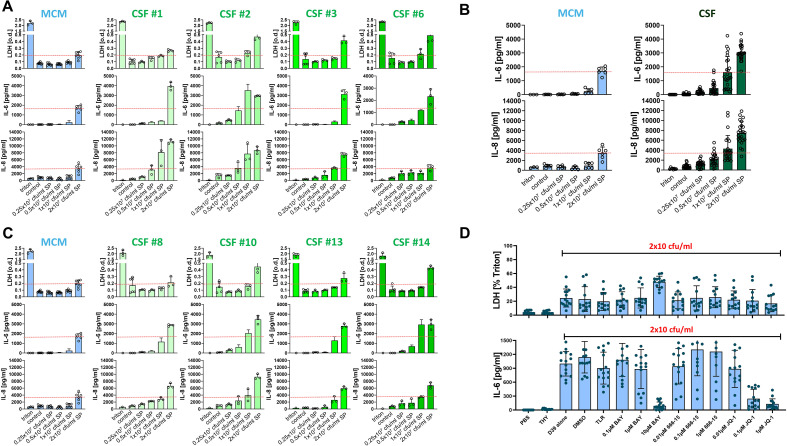
Pneumococcal challenge in human CSF generated higher release of LDH, IL-6 and IL-8 by Ben-Men-1 cells. Ben-Men-1 cells were challenged with increasing concentrations of SP D39 (0.25/0.5/1/2x10^7^ CFU/ml) while being held in human CSF from 8 different donors (n=3-6, control: MCM) for 24h. Negative control was pure bacterial THY growth medium. Supernatants were analyzed for LDH (positive control: triton 1%), IL-6 and IL-8 **(A, C)**. **(B)** = Summary of data from **(A–C)**. During pneumococcal challenge (2x10^7^ CFU/ml, MCM, negative controls: THY medium, PBS), cells were treated with the following inhibitors: TLR-cocktail (25µg/ml anti-TLR2 antibody + 5µM TLR4 inhibitor + 20µg/ml chloroquine), 0.1-10 µM BAY 11-7082, 0.01-1µM 666-15, 0.01-1µM JQ1 or DMSO control. **(D)** Supernatants were analyzed for LDH and IL-6. Data are given as individual values as well as means ± SD.

To examine the role of meningeal fibroblasts in pneumococcal infection, we first employed the meningioma-derived cell line Ben-Men-1, which has been used as a model of meningeal fibroblasts in previous studies (16) and is known to release cytokine upon LPS stimulation. In parallel, we established primary HMC as a novel and more physiologically relevant model. To ensure comparability between both cell types, Ben-Men-1 cells were also cultured in MCM, which is routinely used for HMC. We observed that Ben-Men-1 cells displayed marked differences in phenotype and behavior dependent on culture conditions. When cultured in standard DMEM, cells grew rapidly, formed multilayers, and exhibited a more round morphology, whereas growth in MCM was slower, monolayered, and spindle-shaped (**Figure 2**). Despite these morphological differences, pneumococci-induced cell death (measured by LDHrelease) was comparable between both conditions across a range of bacterial concentrations (0.25x10^7^ to 8x10^7^ CFU/ml). In contrast, cytokine responses differed between culture conditions: IL-6 and IL-8 release occurred at lower pneumococcal concentrations in MCM than in DMEM. These cytokines were selected based on previous reports of their expression in Ben-Men-1 cells upon LPS stimulation (16) and our pilot experiments demonstrating their release upon SP challenge in a protein array (27). Given the observed heterogeneity across culture conditions, we next investigated Ben-Men-1 cell responses in human CSF to better approximate physiological conditions during PM. CSF was chosen because its protein content and nutrient composition more closely reflects the *in vivo* extracellular environment than standard culture media and may therefore influence cellular responsiveness. For these experiments, Ben-Men-1 cells were pre-cultured in MCM and stimulated with SP (0.25/0.5/1/2x10^7^ CFU/ml) in CSF obtained from patients with IIH or NPH (**Supplementary Table 1**). Across all CSF samples, pneumococcal stimulation induced concentration-dependent increases in LDH release as well as IL-6 and IL-8 production, which were generally more pronounced than in MCM. However, inter-individual variability between CSF samples was observed; for example, CSF from patients #1 and #2 induced significantly higher IL-8 release at 1×10^7^ CFU/ml compared to other samples (**Figures 3A, C**).

### 3.2 BRD4 regulates IL-6 release in Ben-Men-1 cells

To identify signaling pathways involved in meningeal fibroblast activation upon pneumococcal exposure, Ben-Men-1 cells were pretreated with selected inhibitors prior to challenge with SP (2×10^7^ CFU/ml).

Among the pathways tested, only inhibition of BRD4 by JQ-1 significantly reduced IL-6 release (**Figure 3D**). The apparent reduction in IL-6 production by the NF-κB inhibitor BAY 11–7082 at the highest concentration was likely attributable to cytotoxicity. Inhibitors targeting CREB and TLR2, TLR4, and endosomal TLRs did not significantly affect inflammatory activation of Ben-Men-1 cells after pneumococcal challenge (**Figure 3D**).

These findings indicate that IL-6 release in Ben-Men-1 cells is predominantly dependent on BRD4-mediated transcriptional regulation.

### 3.3 Primary meningeal fibroblasts exhibit PLY- and NF-κB-dependent activation

**Figure 4:**
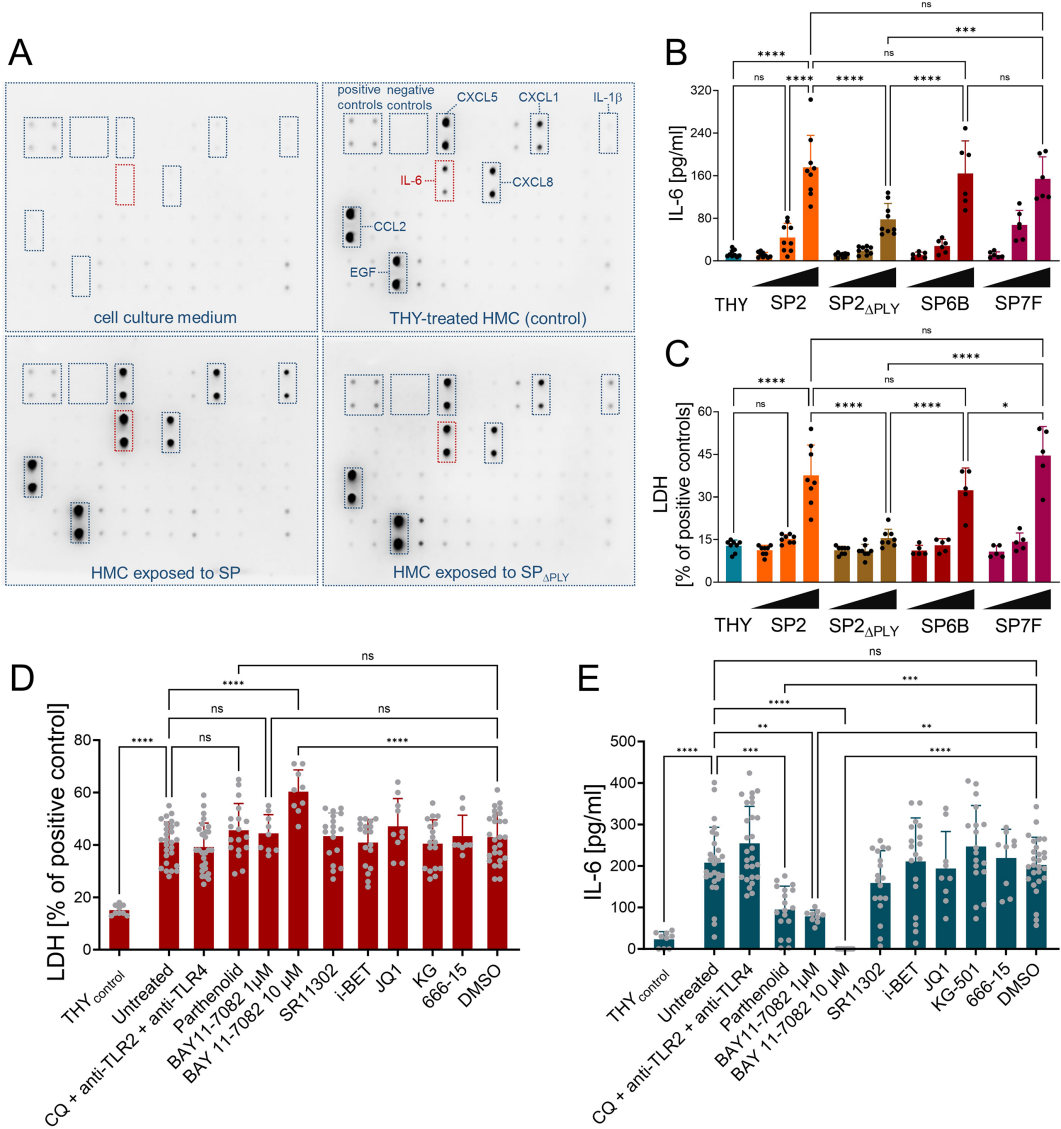
Primary human meningeal cells display partially PLY-dependent cell death and cytokine release upon pneumococcal challenge. HMC were challenged with P/S-lysed *S. pneumoniae* serotype 2 (D39), D39Δ*ply*, serotypes 6B and 7F (0.25/1/4x10^7^ CFU/ml, negative control: THY bacterial medium without bacteria) for 24h. Supernatants were analyzed for LDH **(C, D)** and IL-6 release **(B, E)** After exposure to THY, D39 or D39Δ*ply* (4x10^7^ CFU/ml), supernatants were pooled and analyzed with antibody array. **(A)** HMC were treated during pneumococcal challenge (4x10^7^ CFU/ml) with the following inhibitors: TLR-cocktail (anti-TLR2 antibody T2.5 (25µg/ml) + TLR4 inhibitor TAK-242 (5µM) + endosomal TLR inhibitor chloroquine (20µg/ml)), NF-κB inhibitors parthenolide (0.5µM) and BAY 11-7082 (1-10 µM), AP-1 inhibitor SR11302 (1µM), BRD4 inhibitors I-BET (10µM) and JQ1 (1µM), CREB inhibitors KG-501 (10µM) and 666-15 (1µM), or DMSO control. **(D, E)** Data are given as individual values as well as means ± SD. *p < 0.05, **p < 0.01, ***p < 0.001, ****p < 0.0001. Statistical analysis was calculated with ANOVA with Tukey’s multiple comparison test.

As primary HMC have not previously been studied in the context of pneumococcal infection, we first characterized their responses to challenge with SP using a panel of strains selected to address different experimental aspects. We included the well-established laboratory strain D39 (serotype 2) as a reference, its isogenic PLY-deficient mutant D39Δ*ply* to assess the contribution of PLY, and the clinically relevant serotypes 6B and 7F to account for potential serotype-dependent differences. This selection was based on known differences in pathogenicity, as serotype 6B caused higher mortality and stronger inflammatory cytokine response in the CSF than 7F (28). Pneumococcal challenge induced dose-dependent lytic cell death, which was partially dependent on PLY (**Figure 4C**). In addition, HMC released a distinct set of cytokines and growth factors, including IL-1β, IL-6, IL-8 (CXCL8), CXCL-1, CCL2, and EGF (**Figure 4A**). Cytokine release was partially dependent on the presence of PLY (**Figures 4A, B**).

Compared to Ben-Men-1 cells under identical stimulation conditions, HMC showed similarqualitative response patterns, but differed in the magnitude and variability of cytokine production. Notably, in contrast to Ben-Men-1 cells, IL-6 release in HMC was not affected by BRD4-inhibition, but was partially reduced by the NF-κB inhibitors parthenolide and BAY 11-7082. These findings highlight differences in signaling pathways between transformed and primary meningeal fibroblasts. Moreover, combined inhibition of TLR2, TLR4, and endosomal TLRs (using chloroquine) did not affect SP-induced IL-6 release from HMC, consistent with findings in Ben-Men-1 cells. In our previous work, we demonstrated that TLRs - particularly TLR2 and endosomal TLR8 - are responsible for the recognition of SP by human macrophages (29). Given the lack of effect of TLR inhibition in meningeal fibroblasts, we next assessed TLR expression at the mRNA level to determine whether these receptors are expressed in these cells (**Supplementary Table 2**). Both, HMC and Ben-Men-1 cells expressed fibroblast markers (fibronectin, PDGFR-α and collagen type 1α1 (Col1α1)), but lacked detectable TLR2 and TLR8 expression, with only a weak TLR4-expression. In contrast, THP-1 macrophages exhibited strong TLR2 and weak to moderate TLR4 and TLR8 expression.

### 3.4 Macrophage co-culture enhances fibroblast cytokine release via IL-1β

**Figure 5:**
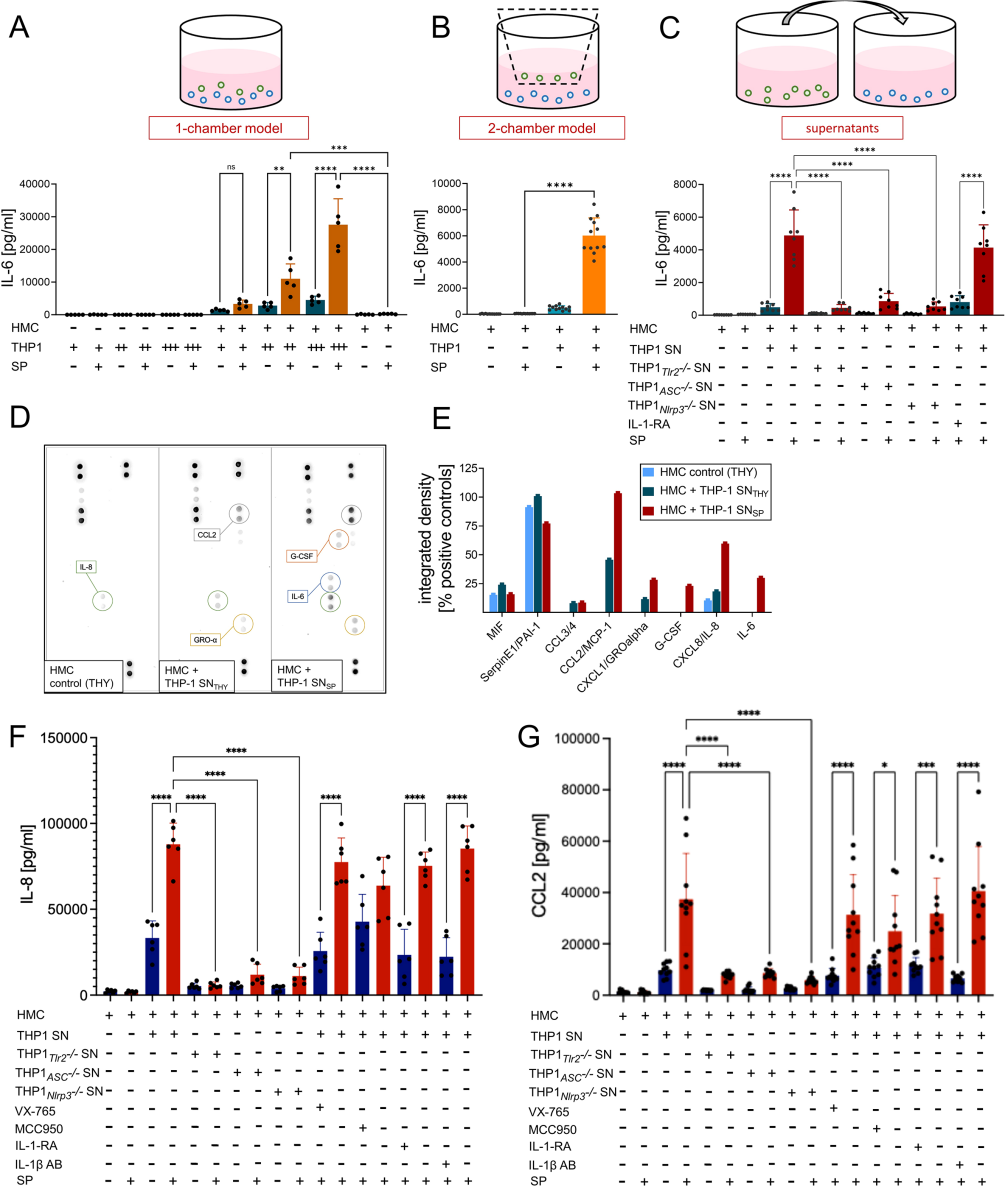
Co-culture with macrophages severely enhances release of IL-6, IL-8 and CCL2 by primary meningeal fibroblasts via IL-1β. HMC were co-cultured with THP-1 macrophages and challenged with SP D39 (3x10^7^ CFU/ml, negative control: THY-medium without bacteria) for 24h. **(A)** direct co-culture in one chamber, B: indirect co-culture with cell types being separated by a membrane with 3µm pores, C-G: indirect co-culture where supernatant (SN) of pneumococci-stimulated THP-1 macrophages (negative control: SN of THP-1 stimulated with THY-medium without bacteria) was added in a 1:10 ratio. After 24h, supernatant of HMC was analyzed for IL-6 **(A-C)**, IL-8 **(F)** and CCL2 **(G)** by ELISA. **(C, F, G)** SN of TLR2^-/--^, ASC^-/--^ and NLRP3^-/--^THP-1 cells were used compared to wild-type THP-1. **(F, G)** THP-1 cells were pretreated with VX-765 (100µM), MCC950 (10µM) or respective control (DMSO, PBS) before pneumococcal stimulation. HMC were pretreated with 5µg/ml IL-1 receptor antagonist, 25µg/ml IL-1β antibody or their respective control additionally to conditioned THP-1 SN before pneumococcal stimulation. Data are given as individual values as well as means ± SD. *p < 0.05, **p < 0.01, ***p < 0.001, ****p < 0.0001. Statistic analysis was calculated with one-way ANOVA. D: Antibody arrays of pooled supernatants of HMC stimulated with control (left), unstimulated THP-1 SN (middle) or SP-stimulated THP-1 SN (right). Upregulated factors are marked by circles. E: Densitometric analysis of fluorescence emission by antibody arrays: for each value, the respective value of negative control was subtracted and indicated as % of positive control. In Panels **(C)**, **(F)**, and **(G)**, the treatment groups were each compared with the corresponding placebo groups, which were treated with the respective solvent instead of the inhibitor.

**Figure 6:**
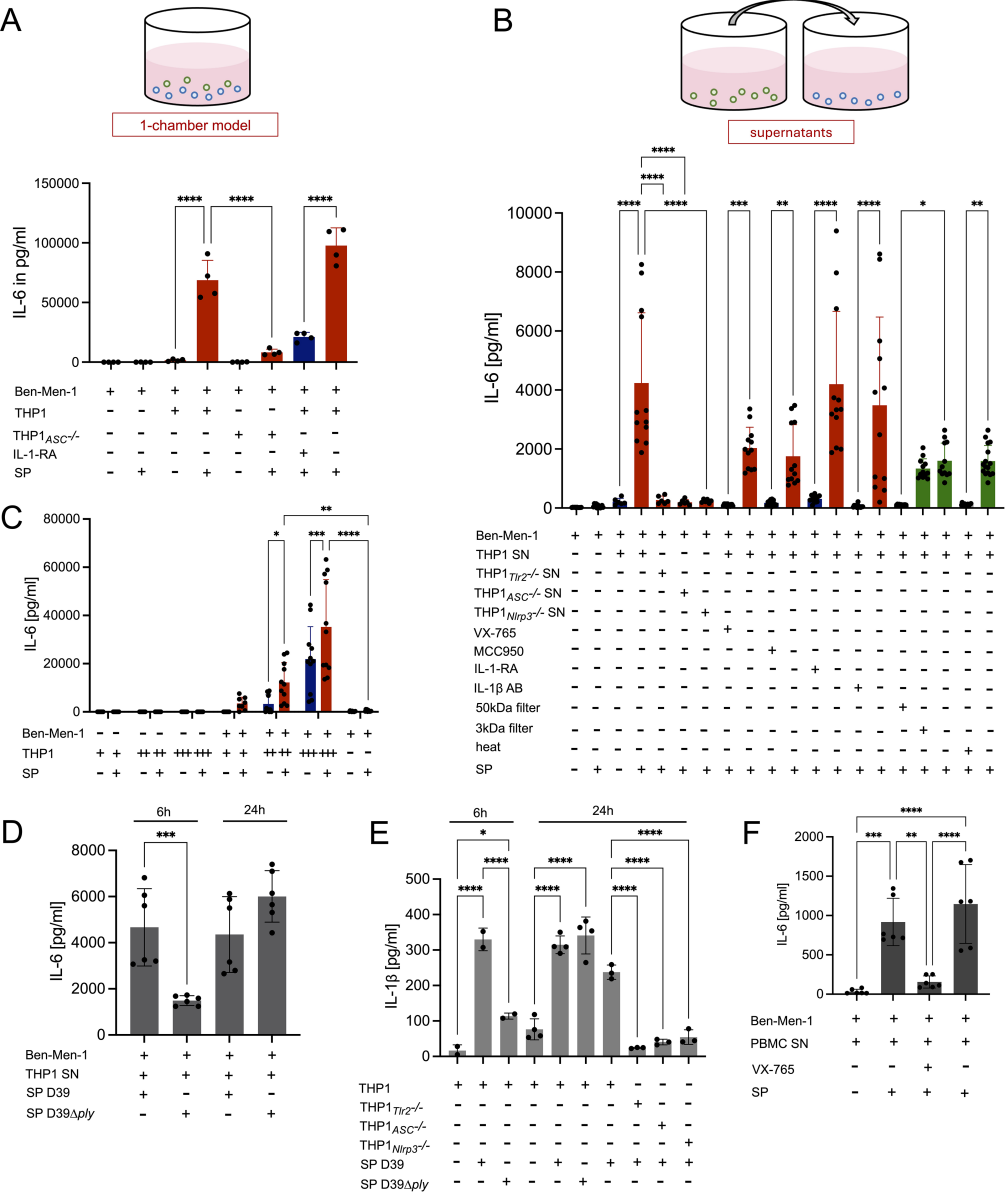
Release of IL-6 by Ben-Men-1 cells upon pneumococcal challenge is increased by macrophage or PBMC IL-1β secretion in direct and indirect co-culture. Ben-Men-1 cells were seeded into PLL-covered plates in MCM and cultivated for 48h. PMA-differentiated THP-1 macrophages were added directly into each well for direct co-culture (**(A)** 30,000 THP-1 cells/well, **(C)** 15,625(+), 62,500 (++) or 250,000 (+++) cells/well). Cells were then stimulated with SP D39 (3x10^7^ CFU/ml, n=4 **(A)**, 4x10^7^ CFU/ml **(C)**, negative control: THY-medium without bacteria) for 24h and IL-6 release determined. For indirect co-culture with supernatant (SN), THP-1 macrophages (WT, TLR2^-/-^, ASC^-/-^ or NLRP3^-/-^) were stimulated with SP D39 or D39Δ*ply* (3x10^7^ CFU/ml, negative control: THY-medium without bacteria) for 6h or 24h **(B, D, E)**. PBMC were isolated from blood samples and stimulated analog to THP-1 for 24h, with or without VX-765 pretreatment **(F)**. Supernatants were harvested, frozen at 30 °C and analyzed for IL-1β release (n=2-4) **(E)** or used to stimulate Ben-Men-1 cells in 24 well plates in a 1:10 ratio. After co-culture stimulation experiments, plates were centrifuged and their supernatants harvested for measuring cytokine production of IL-6 by ELISA. **(B)** SN of TLR2^-/-^, ASC^-/-^ and NLRP3^-/-^ THP-1 macrophages were used compared to WT. Additionally, THP-1 macrophages were pretreated with VX-765 (100µM), MCC950 (10µM) and their respective controls before pneumococcal stimulation, and their supernatant used for indirect co-culture. Moreover, SN was pretreated with 3kDa and 50kDa filters as well as heated to 60 °C for 30 minutes, respectively, before use for indirect co-culture experiments. Ben-Men-1 cells were treated with 5µg/ml IL-1 receptor antagonist or 25µg/ml IL-1β antibody or their respective controls before indirect stimulation with THP-1 SN. Data are given as individual values as well as means ± SD. *p < 0.05, **p < 0.01, ***p < 0.001, ****p < 0.0001. Statistic analysis was calculated with one-way or two-way ANOVA. In Panels **(A), (B), (E)**, and **(F)**, the treatment groups were each compared with the corresponding placebo groups, which were treated with the respective solvent instead of the inhibitor.

To analyze the interactions between macrophages and meningeal fibroblasts, we established multiple co-culture models. Direct co-culture of Ben-Men-1 cells or HMC with THP-1 macrophages followed by SP challenge (3x10^7^ CFU/ml) resulted in a marked increase in IL-6 release by meningeal cells (approximately 600-fold and 80-fold, respectively), with stronger effects at higher macrophage numbers (**Figures 5A**, **6C**). A similar enhancement was observed in indirect co-culture systems, including transwell setups where the different cell types were separated by a pore membrane, and stimulation with conditioned supernatants from SP-challenged macrophages (**Figures 5**, **6**), indicating that a soluble mediator drives fibroblast activation. Treatment of Ben-Men-1 cells with supernatant from SP-stimulated macrophages resulted in strong IL-6 expression. In HMC, conditioned supernatants increased IL-6, IL-8, G-CSF, and CCL2 expression (**Figures 5D, E**).

Biochemical characterization of conditioned supernatants revealed that the stimulatory factor was a heat-labile protein with a molecular weight between 3kDa and 50kDa. Next, we observed that supernatants from TLR2-, ASC-, or NLRP3-deficient macrophages failed to enhance IL-6 release from meningeal cells, indicating dependence on inflammasome activation and suggesting IL-1 family cytokines as candidates. Consistently, pharmacological inhibition of caspase-1 (VX-765) or NLRP3 (MCC950) attenuated fibroblast activation. Using the experimental strategy illustrated in **Figure 1**, we obtained converging evidence that IL-1β is the central mediator: blockade of IL-1 receptor signaling with anakinra – known to inhibit IL-1α and IL-1β, but not IL-18 signaling (30, 31) – significantly reduced cytokine release, and neutralization of IL-1β using a specific antibody (MAB401) markedly attenuated fibroblast activation (**Figures 5**, **6**). In HMC, the enhancing effect of macrophage IL-1β was not only observed for IL-6, but also for IL-8 and CCL2.

Consistent with these findings, IL-1β levels were significantly reduced in supernatants of TLR2^-/-^, ASC^-/-^ and NLRP3^-/-^ THP-1 macrophages compared to WT cells (**Figure 6E**). Moreover, stimulation with a PLY-deficient pneumococcal strain resulted in lower IL-1β levels, explaining its reduced capacity to induce meningeal fibroblasts (**Figures 6D, E**). Finally, the use of ASC-deficient macrophages and an IL-1 receptor antagonist also attenuated fibroblast activation in direct co-culture, confirming the central role of IL-1β across co-culture models (**Figure 6A**).

### 3.5 IL-1β driven IL-6 release by meningeal fibroblasts is also induced by PBMC

To determine whether an IL-1β mediated fibroblast activation extends beyond THP-1 macrophages, we used conditioned supernatants from SP-stimulated PBMC. Similar to macrophage supernatants, conditioned medium from PBMC significantly increased IL-6 release in Ben-Men-1 cells (**Figure 6F**). This effect was abolished by caspase-1 inhibition during PBMC stimulation, indicating that fibroblast activation is mediated by IL-1β. These findings suggest that IL-1β-dependent activation of meningeal fibroblasts represents a broader mechanism of immune-stromal interaction during pneumococcal infection.

## 4 Discussion

In this study, we demonstrate that pneumococcal exposure induces cytokine production in meningeal fibroblasts, particularly IL-6 and IL-8. This response was partially PLY-dependent and largely independent of TLR signaling. Importantly, co-culture with macrophages markedly amplified fibroblast activation, and we identified IL-1β as the key mediator driving this response.

In this context, IL-1β represents a central mediator of inflammation and has been strongly implicated in the pathogenesis of PM. It is generated through NF-κB-dependent transcription of pro-IL-1β followed by inflammasome-mediated processing, involving adapter protein ASC and caspase-1 (32, 33). Binding of IL-1β to IL-1 receptor 1 activates NF-κB and mitogen-activated protein kinases (MAPK) signaling pathways, thereby promoting the expression of pro-inflammatory genes. In the intact CNS, IL-1β is involved in neuronal differentiation and survival, neurite growth, neuroplasticity as well as neuroendocrine functions (32). In experimental PM, inflammasome-dependent IL-1β production contributes to disease severity, whereas its inhibition reduces inflammation and neurological damage (34, 35). Comparative studies indicate that IL-1β, rather than IL-18, is the predominant mediator of inflammasome-driven pathology (34) and IL-1 receptor antagonism attenuates neuroinflammation and functional impairment (36). Consistently, elevated CSF caspase-1 and IL-1β levels correlate with disease severity, while caspase-1 inhibition improves outcome (35, 37, 38). However, IL-1 signaling also contributes to antibacterial host defense, as IL-1R-deficient mice show impaired bacterial clearance and increased mortality (39). Together, these findings argue against indiscriminate systemic blockade of IL-1β during acute PM and instead support targeted modulation of downstream inflammatory amplification. In this context, fibroblast-directed strategy may offer improved selectivity, for example by inhibiting NF-κB–dependent cytokine production. While approaches such as fibroblast-targeted nanoparticle delivery remain experimental, systemic IL-1R antagonism in combination with effective antibiotic therapy may represent a more immediately feasible strategy to limit excessive inflammation without compromising pathogen control.

In line with previous studies (20), we observed PLY-dependent cytotoxicity at high pneumococcal concentrations. However, in contrast to earlier reports (20), we consistently detected cytokine release by meningeal cells following pneumococcal exposure. The discrepancy may be explained by differences in bacterial concentrations and experimental conditions, as lower concentrations were below the threshold for cytokine production in our study, whereas higher concentrations cause lytic cell death and prevent cytokine release. Differences in cell models may further contribute, as Fowler et al. used freshly isolated benign meningioma cells, whereas we employed Ben-Men-1 cells and HMC. Moreover, adaptation of culture conditions and stimulation in human CSF enhanced cytokine responses, highlighting the importance of physiologically relevant environments.

These results are in line with previous studies *in vitro* and *in vivo*, showing that meningeal fibroblasts are potential cytokine producers when treated with LPS or other stimuli (13, 16, 17, 40, 41).

To further dissect the signaling mechanisms underlying SP-induced cytokine production, we targeted pathways known to regulate IL-6 expression. Stimulation with bacterial or viral PAMPs or pro-inflammatory cytokines induces IL-6 expression via activation of transcription factors, including NF-κB, AP-1, and CREB (23–25), with additional regulation by the epigenetic reader BRD4 (26) (**Supplementary Figure 1**). Using pharmacological inhibitors, we found that NF-κB plays an important role in IL-6 production in meningeal fibroblasts. In primary HMC, NF-κB inhibition reduced IL-6 release by more than 50%, consistent with previous observations in macrophages and microglia (42–45) as well as meningeal fibroblasts (16). Despite reaching closer similarity of the phenotype of Ben-Men-1 cells to primary HMC by adapting the culture conditions, the examination of the signaling pathways leading to fibroblast activation and by SP exposure revealed significant differences: While NF-κB inhibition reduced IL-6 production in both models, the effect in Ben-Men-1 cells was only observed at higher concentrations of BAY11–7082 and was associated with increased cytotoxicity, suggesting non-specific effects. In contrast, BRD4 inhibition attenuated IL-6 production only in Ben-Men-1 cells but not in primary HMC, likely reflecting differences between tumor-derived cell lines and primary cells. BRD4 binds to transcription factors and regulates inflammation, chromatin assembly, oxidative stress injury, and cell proliferation (46). Tumor-derived cells or immortalized cell lines often exhibit altered chromatin landscapes with increased transcriptional dependency on BET proteins, which may explain their higher sensitivity to BRD inhibition (47, 48). Consistent with this, BET inhibition has been shown to suppress proliferation and transcriptional activity in CNS tumor cells (49–51), whereas primary HMC may rely more directly on canonical NF-κB signaling.

Consistent with studies in peripheral tissues, we observed strong IL-1β-dependent crosstalk between macrophages and fibroblasts. Similar interactions have been described in inflammatory or tumor contexts, where macrophage-derived IL-1β induces fibroblast production of cytokines such as IL-6 and IL-8 (52–57). Comparable effects have been reported in co-culture systems of fibroblasts and monocytes, as well as in fibroblast-like cells derived from the CSF (58–61). However, such mechanisms have not previously been demonstrated in CNS infection. Our findings therefore extend the concept of fibroblast-immune cell crosstalk to PM and identify meningeal fibroblasts as amplifiers of inflammation.

This study has several limitations. Cytokine levels in primary HMC varied between donors, reflecting inter-individual differences in immune responses. Furthermore, we did not directly investigate meningeal macrophages. However, our findings using PBMC suggest that the crucial factor is the capacity of immune cells to produce IL-1β upon pneumococcal stimulation, a mechanism well established for microglia (62).

In conclusion, we identify a previously unrecognized interaction between macrophages and meningeal fibroblasts, in which macrophage-derived IL-1β drives robust cytokine production by fibroblasts. This crosstalk likely contributes to the excessive inflammatory response observed in PM and may help explain the limited effect of macrophage depletion on the immune response in experimental models (3). Rather than being driven by a single cell type, our data support a model in which interactions between multiple cell populations in the leptomeningeal space promote inflammatory amplification.

Future studies should validate this macrophage-fibroblast interaction *in vivo* using established models of PM and assess the contribution of meningeal fibroblasts to cytokine amplification. Approaches such as fibroblast-specific ablation strategies, e.g. Col1a2-Cre lines combined with inducible toxin systems (63) and analysis of human leptomeningeal tissue may provide complementary evidence for the relevance of this pathway (64). In addition, more advanced CNS model systems, including leptomeningeal neural organoid fusion models (65), may enable more detailed mechanistic studies under more physiological conditions.

## Data Availability

The raw data supporting the conclusions of this article will be made available by the authors, without undue reservation.

## Supplementary material

Supplementary Figure 1 Overview of signaling pathways and inhibitors used to assess IL-6 regulation. Schematic representation of TLR- and IL-1 receptor–mediated signaling via MyD88 leading to activation of NF-κB, AP-1, and CREB, which regulate IL-6 transcription. BRD4 acts as a transcriptional coactivator. Pharmacological inhibitors targeting these pathways are indicated.

Supplementary Table 1 Demographic and laboratory characteristics of CSF donors. CSF samples from patients who had undergone CSF drain due to IIHor suspected NPH were analyzed for IL-6 and IL-8 by ELISA. Information on gender, age, diagnosis and clinical laboratory parameters (CSF cell count, CSF total protein, CSF and blood glucose) were obtained from patients’ files. LP, lumbar puncture.

Supplementary Table 2 mRNA expression of selective TLR and fibroblast markers in meningeal fibroblasts and macrophages. qPCR was performed using primers for TLR2, TLR4, TLR8 and fibroblast markers fibronectin (FN), collagen type 1α1 (Col1α1) and PDGFR-α in Ben-Men-1 cells cultured in different media, HMC and THP-1 macrophages. Values are given in relation to housekeeping gene GAPDH. Stable GAPDH expression is shown by highly consistent Ct values (right column).
