# Supplementary figures and images for: Macrophage IL-1β turns meningeal fibroblasts into inflammatory amplifiers in pneumococcal infection
Source: Front Immunol. 2026 May 14;17:1808185. doi: 10.3389/fimmu.2026.1808185 (PMC13215843; doi:10.3389/fimmu.2026.1808185)

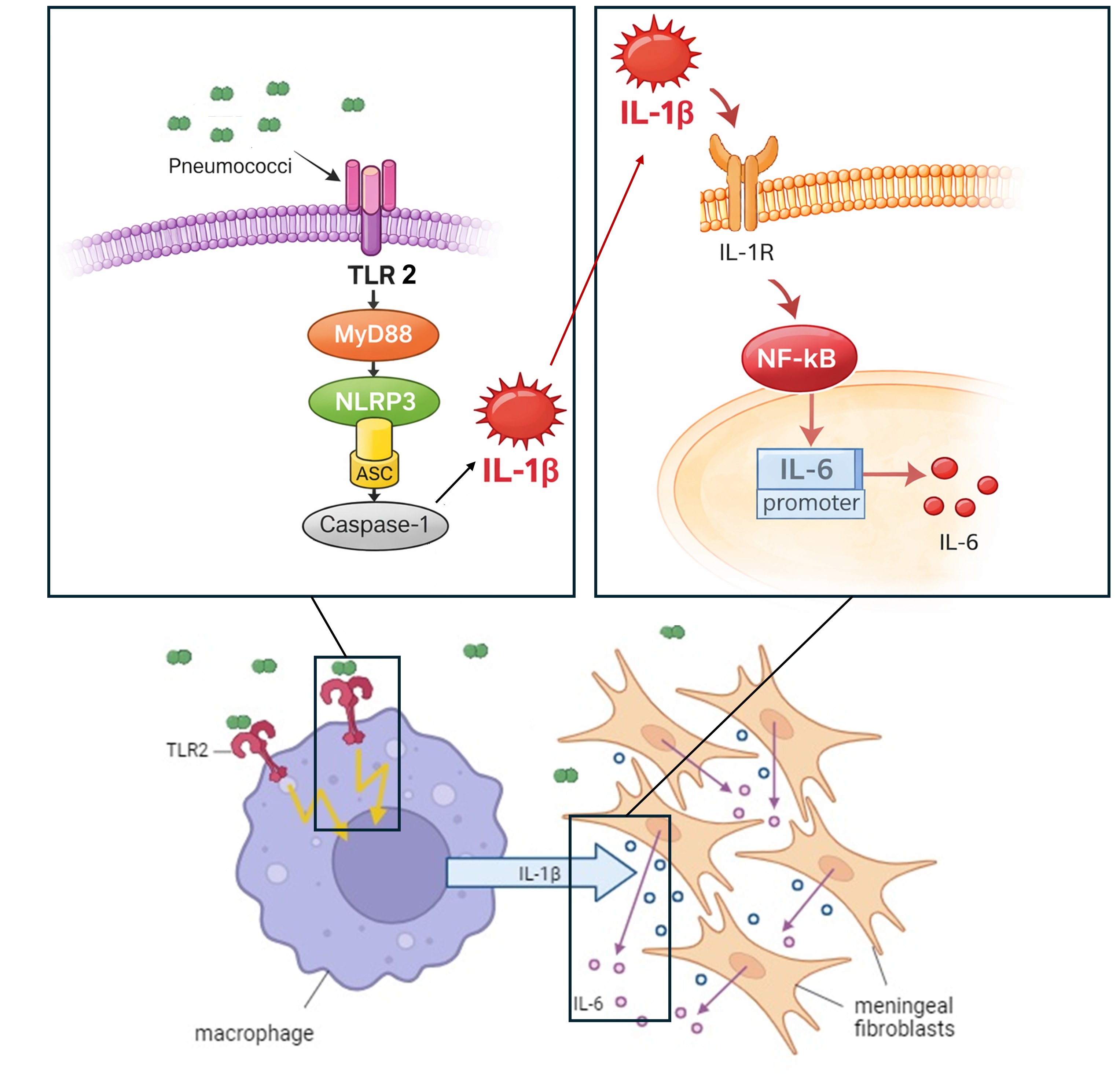

Supplement: Supplementary Figure 1 — Overview of signaling pathways and inhibitors used to assess IL-6 regulation. Schematic representation of TLR- and IL-1 receptor–mediated signaling via MyD88 leading to activation of NF-κB, AP-1, and CREB, which regulate IL-6 transcription. BRD4 acts as a transcriptional coactivator. Pharmacological inhibitors targeting these pathways are indicated. [file Image1.jpeg]

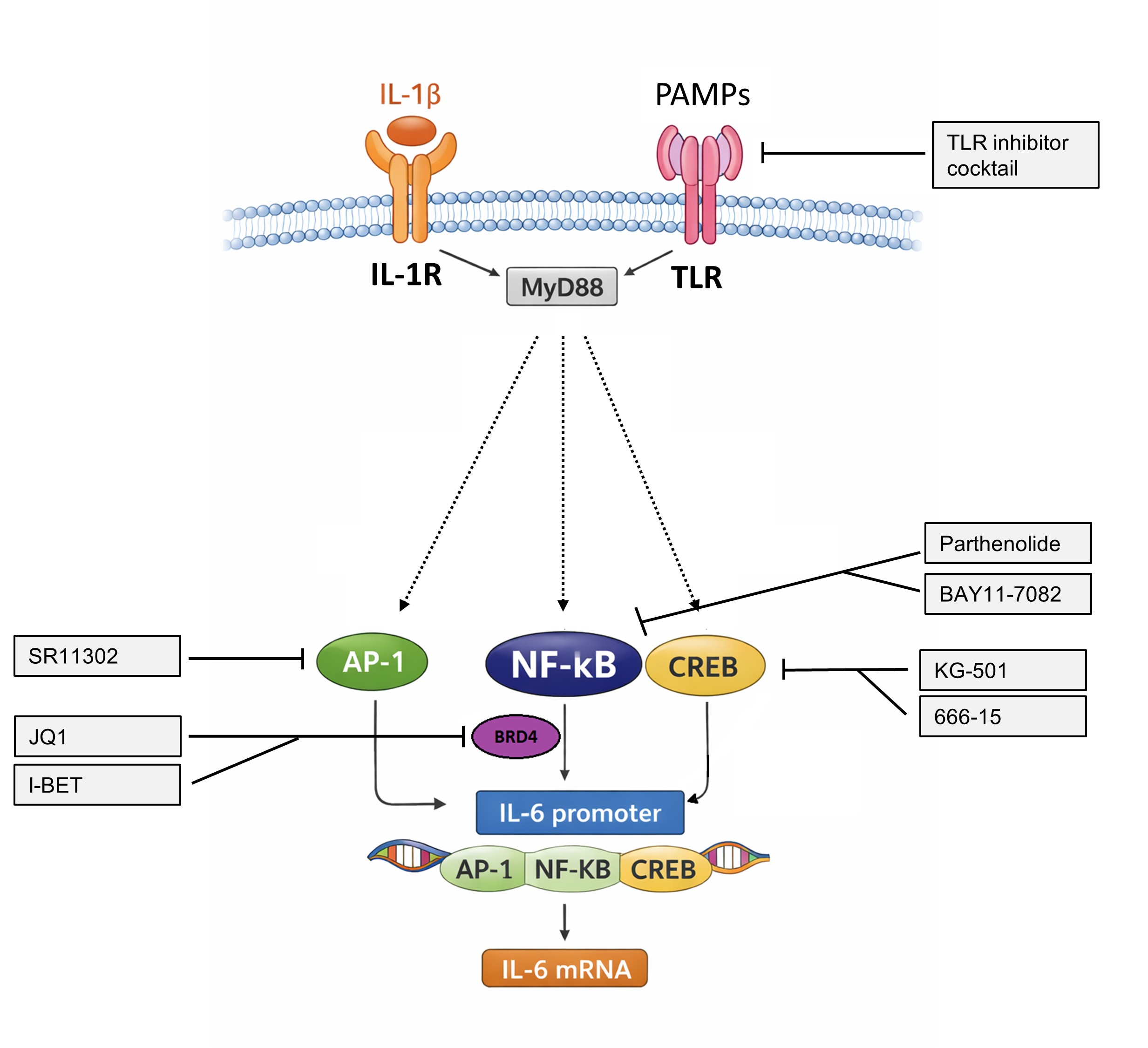

Supplement: Supplementary file 2 [file Image2.jpeg]
