# Supplementary material for: Macrophage IL-1β turns meningeal fibroblasts into inflammatory amplifiers in pneumococcal infection
Source: Front Immunol. 2026 May 14;17:1808185. doi: 10.3389/fimmu.2026.1808185 (PMC13215843; doi:10.3389/fimmu.2026.1808185)
Supplement: Supplementary Table 1 — Demographic and laboratory characteristics of CSF donors. CSF samples from patients who had undergone CSF drain due to IIHor suspected NPH were analyzed for IL-6 and IL-8 by ELISA. Information on gender, age, diagnosis and clinical laboratory parameters (CSF cell count, CSF total protein, CSF and blood glucose) were obtained from patients’ files. LP, lumbar puncture. [file Table1.docx]

| **CSF No.** | **#1** | **#2** | **#3** | **#6** | **#8** | **#10** | **#13** | **#14** |
| --- | --- | --- | --- | --- | --- | --- | --- | --- |
| **Gender** | M | M | M | F | F | M | F | F |
| **Age at LP** | 79 | 84 | 83 | 78 | 51 | 65 | 23 | 34 |
| **Diagnosis** | NPH | NPH | NPH | NPH | PC | NPH | PC | PC |
| **CSF cell count /µl** | 0 | <1 | <1 | 1 | 4 | 2 | 1 | <1 |
| **CSF protein (mg/dl)** | 45 | 57 | 40 | 42 | 46 | 78 | 12 | 74 |
| **CSF glucose (mg/dl)** | 61 | 67 | 72 | 67 | 55 | 67 | 69 | 74 |
| **Blood glucose (mg/dl)** | 97 | 94 | n.d. | 107 | n.d. | 107 | 103 | 117 |
| **IL-6 (pg/ml)** | 0 | 2.5 | 0 | 2.6 | 0 | 0 | 0 | 0 |
| **IL-8 (pg/ml)** | 180.3 | 186.3 | 204.4 | 267.3 | 590.2 | 308.4 | 305.0 | 90.2 |
