# Supplementary material for: Macrophage IL-1β turns meningeal fibroblasts into inflammatory amplifiers in pneumococcal infection
Source: Front Immunol. 2026 May 14;17:1808185. doi: 10.3389/fimmu.2026.1808185 (PMC13215843; doi:10.3389/fimmu.2026.1808185)
Supplement: Supplementary Table 2 — mRNA expression of selective TLR and fibroblast markers in meningeal fibroblasts and macrophages. qPCR was performed using primers for TLR2, TLR4, TLR8 and fibroblast markers fibronectin (FN), collagen type 1α1 (Col1α1) and PDGFR-α in Ben-Men-1 cells cultured in different media, HMC and THP-1 macrophages. Values are given in relation to housekeeping gene GAPDH. Stable GAPDH expression is shown by highly consistent Ct values (right column). [file Table2.docx]

| **Cells** | **TLR2** | **TLR4** | **TLR8** | **FN** | **Col1α1** | **PDGFR-α** | **GAPDH** (Ct value: mean±std) |
| --- | --- | --- | --- | --- | --- | --- | --- |
| **Ben-Men-1 (DMEM)** | 0 | 1 | 0 | 28 | 4 | 2 | 12.9 ± 0.6 |
| **Ben-Men-1 (MCM)** | 0 | 1 | 0 | 44 | 1 | 1 | 12.9 ± 1.0 |
| **Ben-Men-1**  **(CSF)** | 0 | 1 | n.a. | 136 | 10 | 6 | 12.5 ± 0.7 |
| **HMC** | 0 | 2 | 0 | 311 | 50 | 12 | 12.9 ± 0,8 |
| **THP-1** | 11 | 0.7 | 0.1 | 0.1 | n.a. | n.a. | 13.1 ± 0.7 |
